# Purification of Liquid Fraction of Digestates from Different Origins—Comparison of Polymeric and Ceramic Ultrafiltration Membranes Used for This Purpose

**DOI:** 10.3390/membranes14100203

**Published:** 2024-09-25

**Authors:** Agnieszka Urbanowska

**Affiliations:** Department of Water, Wastewater and Waste Technology, Faculty of Environmental Engineering, Wroclaw University of Science and Technology, Wybrzeze Wyspianskiego 27, 50-370 Wroclaw, Poland; agnieszka.urbanowska@pwr.edu.pl

**Keywords:** digestate, municipal waste biogas plant, agricultural biogas plant, membrane processes, polymeric membranes, ceramic membranes, integrated processes

## Abstract

Circular economy, clean technologies, and renewable energy are key to climate protection and modern environmental technology. Recovering water and valuable minerals from the liquid fraction of digestate is in line with this strategy. Digestate, a byproduct of anaerobic methane fermentation in biogas plants, is a potential source of water, minerals for fertilizers, and energy rather than waste. This study examined digestate from municipal and agricultural biogas plants and highlights the need for research on both due to their differences. The use of membrane techniques for water recovery from liquid digestate offers an innovative alternative to conventional methods. This study used standalone membrane filtration and an integrated system to produce water suitable for agricultural use. Ceramic membranes with cut-offs of 1, 5, 15, and 50 kDa and polymeric membranes of polyethersulfone and regenerated cellulose with cut-offs of 10 and 30 kDa were tested. The results showed that the membrane material significantly affects the transport and separation properties. Higher cut-off values increased permeate flux across all membranes. Ceramic membranes were more susceptible to fouling in standalone ultrafiltration, but were more effective in purifying digestate than polymeric membranes. The best results were obtained with a ceramic membrane with a 1 kDa cut-off (for example, for the integrated process and the municipal digestate, the retention rates of COD, BOD_5_ and DOC were 69%, 62%, and 75%, respectively).

## 1. Introduction

The combined effects of demographic growth, the evolving lifestyle of the consumer, economic and industrial development, and climate change have resulted in significant challenges to ensure sufficient and sustainable water resources for municipal and industrial use, as well as agricultural production in many regions of the world [1]. This places a significant burden on present and future generations with regard to the responsibility for the natural environment and the restoration of the ecological balance [2]. The current limitation in the availability of nonrenewable raw material resources is causing a change in environmental protection strategies towards the development of so-called clean production [3]. This can be achieved, for instance, by reducing the amount of pollutants generated or eliminating them entirely, and by producing reusable products. According to the principles of clean production, waste should be transformed into a substrate for a new full-value product [4]. In municipal and agricultural biogas plants, the underlying principles include the use of the methane fermentation process to generate green energy (biogas) and the optimization of the recovery of nutrients from the byproducts of this process [5]. Digestate, the byproduct of the fermentation process in both types of biogas plants, represents a significant environmental concern [6]. The digestate primarily comprises undigested organic matter and mineral components. The aforementioned components are present in quantities comparable to their content in the substrates used in the biogas plant [7]. The quantity of digestate produced is estimated to be in the range of 85–95% of the weight of the substrates employed [8]. The proportion of digestate produced is inversely proportional to the degree of fermentation of the organic matter present in the raw material [9]. The quality of digestate also depends on the source of the microbial inoculum, the conditions under which the fermentation process occurred (pH, temperature, organic matter loading rate, hydraulic retention time), and the type of biogas plant and the type of technology used [10]. Furthermore, pretreatment of input components also influences the physicochemical composition of the final byproduct [11].

It has been incumbent upon municipal facilities to manage and process the digestate primarily through dewatering, drying, and thermal incineration [12]. In contrast, agricultural biogas plants are faced with the challenge of excess digestate production and inadequate management [13]. In practice, this frequently manifests itself as excessive infiltration into soils, resulting in an excess of nutrients and, on occasion, their unintended infiltration into groundwater. Consequently, there is a growing interest annually not only in biogas facilities themselves but also in the processing of digestate, as the management of hydrated digestate gives rise to both logistical and legal issues [14]. For example, it is necessary to store the material for approximately six months in lagoons covered with a geomembrane or in special reinforced concrete tanks, since the decomposition processes are still ongoing [15]. Furthermore, the storage of hydrated digestate requires significant financial investment in the construction of reinforced concrete tanks or, in the case of lagoon construction, the acquisition of an additional land area of approximately 2 to 3 hectares [16]. A study [17] estimated that the costs associated with transporting hydrated digestate over distances greater than 10 km from a biogas plant, due to the significant amount of diesel fuel consumed, begin to exceed the profits associated with its sale as a fertilizer source. Therefore, it can be seen that a solution to the problem of high transport costs, or in the case of digestate storage, to the reduction in the volume of the tanks (lagoons), could be the separation of the digestate into a solid and a liquid fraction. It should be noted that the liquid fraction usually represents between 90 and 95% of the total digestate mass [18]. Therefore, it appears that the concept of digestate management could represent an opportunity for numerous small and medium enterprises that could be involved at this stage in the realization of municipal and agricultural biogas plants.

Given that digestate is treated not only as an alternative fertilizer but also as a source of water, it is essential that it undergoes a process of treatment that ensures that any contaminants present in the fermented biomass are not returned to the environment. In particular, ultrafiltration (UF) represents a potentially valuable technique in this context [19]. The process involves the retention of fine suspended solids, colloids, bacteria, and viruses. The transport mechanism is sieve-like, whereby particles larger than the diameter of the pores are excluded from passing through the membrane. The transmembrane pressure employed falls in the range of 0.1 to 1.0 MPa. The membranes employed in the UF process can be constructed from organic or inorganic materials. Organic membranes include polymeric membranes, comprising materials such as polysulfone, polyethersulfone, polyamide, and cellulosic materials. These membranes have been used in a multitude of applications in a range of fields [20,21]. However, their primary limitation is their limited chemical stability in aggressive systems, including aqueous solutions with low or high pH and solutions containing organic solvents. Nevertheless, they are distinguished by their ease of material processing, relatively low cost, and extensive range of properties. An alternative to polymeric membranes is ceramic membranes derived from inorganic materials. Inorganic membranes are distinguished from their organic counterparts by their high thermal, chemical, and mechanical resistance, as well as their capacity to withstand prolonged use [22]. Ceramic membranes produced from metal oxides, including Al, Ti, and Zr, have been implemented in the widest range of applications. Asymmetric ceramic membranes are made up of multiple layers. The support layer has a thickness of a few millimeters and comprises pores with a diameter of 1–10 µm. The intermediate layer, which has a thickness of 10–100 µm, comprises pores of a larger size, with a diameter of 50–100 µm. The resolution layer, which is the epidermal layer, is the thinnest, with a thickness of approximately 1 µm, and comprises the smallest pores, with a diameter in the range of 2 to 50 nm [23].

The utilization of membrane processes, including ultrafiltration (UF), for the purification of liquid digestate is expected to become increasingly common in the near future. This is due to the enhancement of membrane performance and the reduction in the costs of both membranes and membrane plants. At present, there is a paucity of literature on this subject, with only a small number of reports [24,25,26] available. Moreover, to the best of my knowledge, there are no published studies examining the applications of the aforementioned integrated process configuration for the treatment of digestates derived from diverse sources. The high inconvenience of digestate management, irrespective of its origin, has led to a growing interest in not only improving waste treatment technologies in biogas plants but also in developing new methods of digestate management and in enhancing existing techniques. A study was, therefore, conducted to compare the transport and separation properties of the tested membranes, which were made of organic and inorganic materials, in both a standalone membrane filtration process and an integrated process combining conventional treatment processes with an ultrafiltration process used to treat the liquid digestate fraction from different sources.

In consideration of the presented information, the comparison between the properties of the various membranes is original and has the potential to be highly beneficial in the selection of an appropriate method for the purification of the digestate.

## 2. Materials and Methods

This study employed two distinct types of liquid digest fraction, originating from a municipal biogas plant and an agricultural biogas plant, respectively. The first sample was obtained from a biogas plant processing the organic fraction of municipal waste located in the Lower Silesia Province (Poland, 50°53′15.5″ N 17°23′28.0″ E). The aforementioned fraction was separated from the digestate pulp by means of sedimentation centrifuges. The second fraction was obtained from an agricultural biogas plant located within the Silesian Voivodeship (Poland, 50°17′28.7″ N 18°32′50.3″ E). The samples were taken from a specific point of collection at the outlet of the anaerobic digestion (AD) reactor, prior to the mechanical dewatering installation. The characteristics of the two test solutions are presented in Table 1. The physicochemical analysis of the solutions was conducted in accordance with the Standard Methods for the Examination of Water and Wastewater, 23rd edition [27]. The details of the analytical procedures conducted and the instrumentation used are presented in Table 2.

The UF flat membranes used in the study were four polymeric membranes from Mann + Hummel Water & Fluid Solutions and four ceramic membranes from Tami Industries. The properties of the membranes used are presented in Table 3.

**Figure 1:**
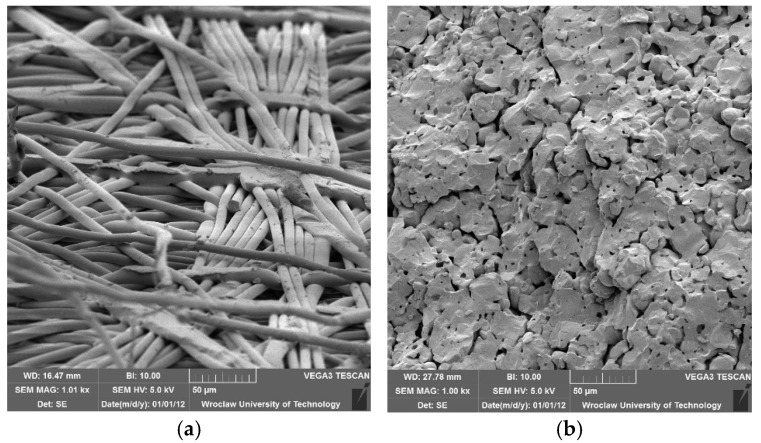
SEM images of selected membranes (1000× magnification): (**a**) polymeric regenerated cellulose 10 kDa, (**b**) ceramic 5 kDa.

The tested membranes were distinguished by a distinctive asymmetric structure, comprising a thin epidermal layer and a thicker support layer. Figure 1 depicts microscopic images of surfaces of selected membranes.

**Figure 2:**
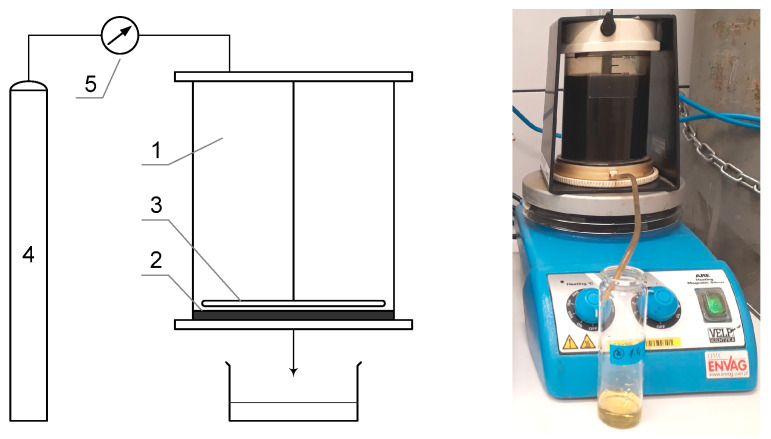
Laboratory setup with Millipore’s Amicon 8400 chamber (1—Amicon 8400 chamber, 2—membrane, 3—stirrer, 4—compressed nitrogen cylinder, 5—regulator).

**Figure 3:**
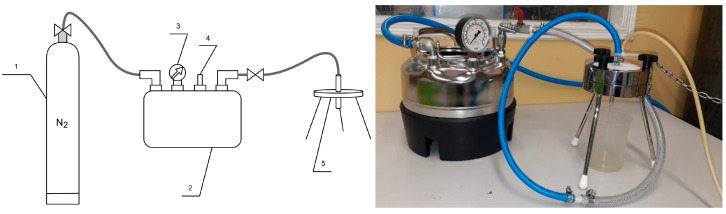
Sterlitech laboratory installation (1—compressed nitrogen cylinder, 2—pressure vessel, 3—manometer, 4—safety valve, 5—ceramic diaphragm in housing).

The pressure membrane filtration process was carried out using two installations, each equipped with a different type of membrane. The digestate was subjected to a 72 h sedimentation period prior to testing. The efficacy of the treatment of the liquid fraction of the digestate using polymeric membranes was evaluated through studies conducted on a test rig equipped with an Amicon 8400 chamber from Millipore (Figure 2). This chamber is designed to accommodate a dead-end filtration process and is intended for use with flat membranes. The Amicon 8400 chamber has a volume of 400 cm^3^ and a membrane diameter of 76 mm. The UF chamber was positioned on an ARE magnetic stirrer from OMC Envag to guarantee that the concentrations were uniform throughout the solution volume. Additionally, experiments using ceramic membranes were conducted using a Sterlitech laboratory apparatus, consisting of a 316 SS pressure chamber with a capacity of 3.8 dm^3^ (Figure 3). This apparatus is designed for operation with flat ceramic membranes in a dead-end configuration. The transmembrane pressure used in the trials was 0.3 MPa. All experiments were performed in duplicate to confirm the obtained results.

Studies on the purification of liquid digestate in an integrated process, which is a combination of conventional purification processes and membrane filtration, were carried out using the installations/test sites described above. Raw digestate was subjected to 72 h sedimentation and purification by filtration using a 1 µm pore size bag filter made of polyester felt material from ChemTech (Poland). The liquid was then coagulated with FeCl_3_⋯6 H_2_O (Avantor Performance Materials Poland S.A., Gliwice, Poland) at 10 g/dm^3^. The coagulation process was carried out by rapid stirring (150 rpm) for 2 min, then the stirrer speed was reduced to about 20 rpm and slow stirring was carried out for 20 min. Standalone coagulation lowered COD, BOD_5_, and DOC to 20,640 g O_2_/m^3^, 6950 g O_2_/m^3^, and 6820 g C/m^3^ for municipal digestate and 28,260 g O_2_/m^3^, 10,370 g O_2_/m^3^, and 17,240 g C/m^3^ for agricultural digestate. The samples were then subjected to sedimentation and filtration using medium tissue filters for 30 min. The resulting clarified liquid was fed to both polymer and ceramic UF membranes.

Before carrying out the membrane filtration process, the polymeric membranes to be tested were subjected to conditioning, which consisted of filtering redistilled water through the membranes successively at different transmembrane pressures ranging from 0.1 to 0.4 MPa. The treatment was carried out until constant flux values were obtained.

The ceramic membranes, on the other hand, underwent a procedure to prepare them for proper operation before being used in the tests. This included alkaline cleaning by placing the membranes in NaOH solution (15–20 g/dm^3^) at 80 °C for 30 min, rinsing until neutral pH, then acid cleaning and rinsing again until neutral pH and initial water flux was restored.

After each experiment, both polymeric and ceramic membranes were cleaned (chemically regenerated) with 0.1 mol/dm^3^ NaOH solution (Avantor Performance Materials Poland S.A., Poland) and washed with redistilled water until the initial permeate flux values were obtained.

The transport properties of membranes used in UF were assessed by determining the permeate flux (J). Volumetric permeate flux is the volume of permeate obtained from a unit membrane surface area per unit time in a membrane process:

(1) $$J=\frac{V}{A\cdot t},\;\frac{m^{3}}{m^{2}\cdot d}$$

where

- V—volume of permeate, m^3^;
- A—membrane surface area, m^2^;
- t—filtration time, d.

Relative membrane permeability values (J/J_0_), calculated as the permeate flux (J) divided by the redistilled water flux of the new membrane (J_0_), were used to characterize membrane fouling intensity.

The selectivity of the UF process was determined by the retention factor of the substance contained in the feed, also known as the reduction/removal ratio (R), according to the following formula:

(2) $$R=\left(1-\frac{c_{p}}{c_{n}}\right)\;\cdot \;100,\;\%$$

where

- c_p_—concentration of impurities in the treated solution, g/m^3^;
- c_n_—initial concentration of impurities in the solution to be purified, g/m^3^.

The values R > 90% were determined with an error of less than 1%.

## 3. Results

### 3.1. Transport Properties of Membranes

**Figure 4:**
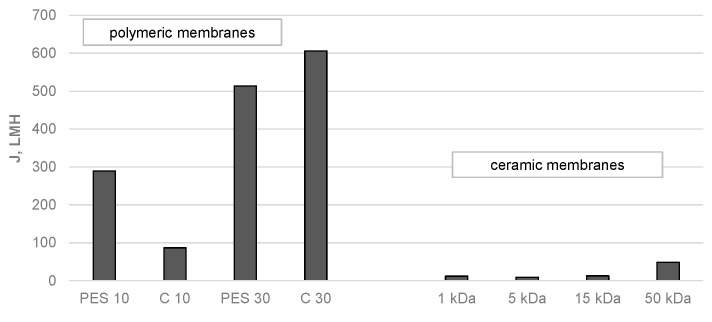
Redistilled water flux for polymeric and ceramic membranes.

When deciding on the suitability of individual membranes for the purification of the test solution, attention should be paid not only to their separation properties but also to their transport properties. The results of the redistilled water flux measurements for the organic and inorganic membranes tested are shown in Figure 4. They show that the hydraulic performance of a membrane depends significantly on the cut-off and the membrane material. For all membranes tested, the redistilled water flux increased with increasing cut-off values. Of all the polymeric membranes tested, the regenerated cellulose membrane with a cut-off of 30 kDa had the highest permeability (605.8 LMH). For a membrane with the same cut-off, but made of polyethersulfone, the permeate flux was 513.8 LMH. Of the ceramic membranes tested, the highest permeate flux, 48.3 LMH, was observed for the 50 kDa membrane. From the results obtained, it can be concluded that the hydraulic permeability of the polymeric membranes was much higher than that of the ceramic membranes.

**Figure 5:**
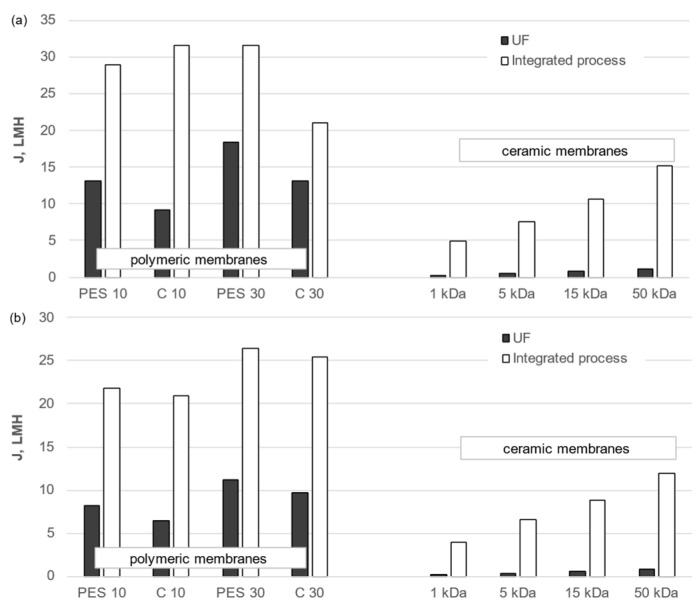
Effect of membrane type and cut-off value on permeate flux for the liquid fraction of municipal (**a**) and agricultural (**b**) digestate.

A similar relationship to that for redistilled water was observed for all membranes used to filter the liquid fraction of both municipal and agricultural digestate (Figure 5)—membranes with higher cut-offs achieved higher permeate fluxes. This trend was observed regardless of whether a standalone UF process was used or whether it was preceded by pretreatment. The permeate flux values for both digestion fluids were also observed to be lower than those for redistilled water. This was due to an increase in flow resistance values due to membrane fouling. It was also observed that the greater the cut-off value of the membranes, the greater the decrease in permeate flux compared to redistilled water, indicating that internal fouling was the dominant factor in their case, caused by the penetration of impurities into the membrane pores.

Furthermore, the content of the treated solution was found to exert a considerable influence on the membrane permeability. Filtration (either standalone or preceded by pretreatment) of the liquid fraction of agricultural digestate resulted in permeate fluxes that were inferior to those observed for the liquid fraction of municipal digestate. This indicates that contaminants present in agricultural digestate, by inducing alterations in the spatial configuration of organic matter and modifying its chemical properties, may intensify the membrane fouling phenomenon.

**Figure 6:**
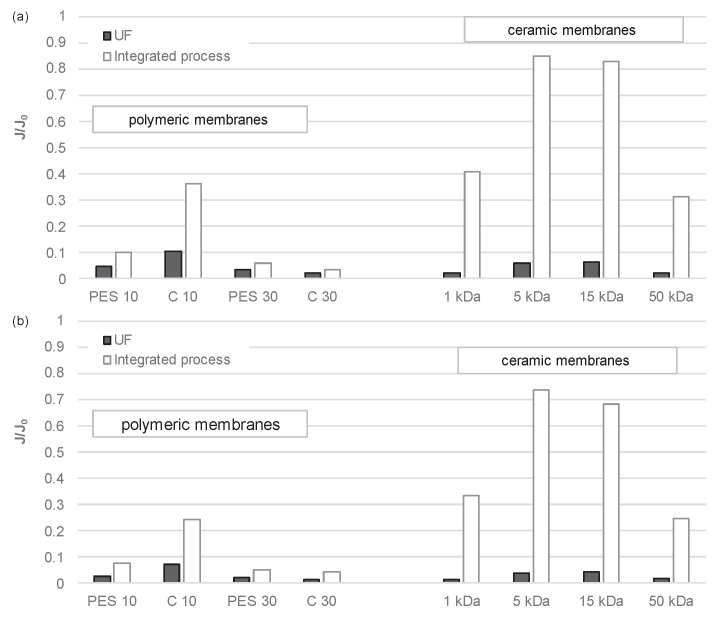
Relative permeability during standalone UF and during the integrated process: sedimentation/filtration/coagulation/sedimentation/UF of (**a**) the liquid fraction of municipal digestate and (**b**) the liquid fraction of agricultural digestate using polymeric and ceramic membranes.

The membranes tested exhibited differences not only in their absolute hydraulic capacity but also in their susceptibility to fouling. Membrane fouling represents a significant challenge in the operation of membrane systems. It arises from the deposition of contaminants from the treated solution on the membrane surface and within the membrane structure. This phenomenon leads to a reduction in the permeate flux (in processes conducted at constant transmembrane pressure) or an increase in pressure (in processes conducted at constant permeate flux). As illustrated in Figure 6, the comparative permeability data (J/J_0_) for the two digestate fluids under examination unambiguously demonstrate that the ceramic membranes exhibited a greater propensity for fouling than the polymeric membranes, particularly those with a cut-off of 1 and 50 kDa. This is likely attributable to their increased hydrophobicity, which results in a more pronounced reduction in permeability. The J/J_0_ values of ceramic membranes with a cut-off of 5 kDa and 15 kDa are comparable to those obtained using regenerated cellulose membranes with a cut-off of 10 kDa and polyethersulfone membranes with a cut-off of 30 kDa, with a value of approximately 0.06. The membrane exhibiting the least degree of blockage was identified as an organic membrane of regenerated cellulose with a cut-off value of 30 kDa (J/J_0_ ≅ 0.07). This can be attributed to the membrane’s notable hydrophilicity, which may have diminished the impact of the physicochemical characteristics of the filtered digestate on membrane fouling. The use of hydrophilic membranes serves to diminish the accumulation of material at the solution–membrane interface and to extend the duty cycle of the membrane. The outcomes align with the documented wetting angles of the membranes (Table 3), substantiating the hydrophilic or hydrophobic nature of the membranes. Membranes with an identical cut-off value, yet manufactured from disparate materials, also exhibit variation in their average pore size, which exerts an influence on the extent of membrane fouling. Irrespective of the membrane type subjected to examination, the resulting agglomerates of organic compounds formed a filter cake on the membrane surface. Similar findings were observed when comparing the fouling intensity of organic and inorganic membranes in an integrated process, which included sedimentation, filtration, coagulation, sedimentation, and UF. Subsequently, it was discovered that polymeric membranes exhibited a greater susceptibility to fouling than ceramic membranes. The J/J_0_ values for the liquid fraction of municipal digestate were 0.10 and 0.06 for polymeric membranes with cut-offs of 10 kDa and 30 kDa, respectively. These values were observed for PES membranes, while for C membranes, the values were 0.36 and 0.03, respectively. In contrast, the values were significantly higher for ceramic membranes, with the highest value observed in membranes with a cut-off of 5 kDa (0.85). For the liquid fraction of agricultural digestate, the corresponding values were 0.07 and 0.05 for PES membranes, 0.24 and 0.04 for C membranes, and 0.74 for the 5 kDa ceramic membrane, respectively. The findings indicate that the utilization of a pretreatment process involving FeCl_3_⋯6 H_2_O had a pronounced impact on the extent of membrane fouling. The purification of the liquid fraction of fermentation in the integrated process, as analyzed in all cases, resulted in a notable reduction in the intensity of membrane fouling. The preceding pretreatment of the solution, thus, results in a notable reduction in the fouling of the membranes under analysis. This was particularly evident when sedimentation, filtration, coagulation, and sedimentation processes were combined in a sequence using a ceramic membrane with a cut-off of 5 or 15 kDa. The results suggest that, in the case of more compact membranes, the use of coagulation preceded by sedimentation and filtration allows the removal of compounds from the digestate that, in the absence of pretreatment, would otherwise settle on the membrane surface and block it. The results obtained for a membrane with a cut-off of 1 kDa represent an exception to this rule. The aforementioned filtration processes on other ceramic membranes also improve the transport properties of the membranes, although to a lesser extent. This may be attributed to the penetration of residual fractions in solution into the membrane pores and their subsequent blocking.

**Figure 7:**
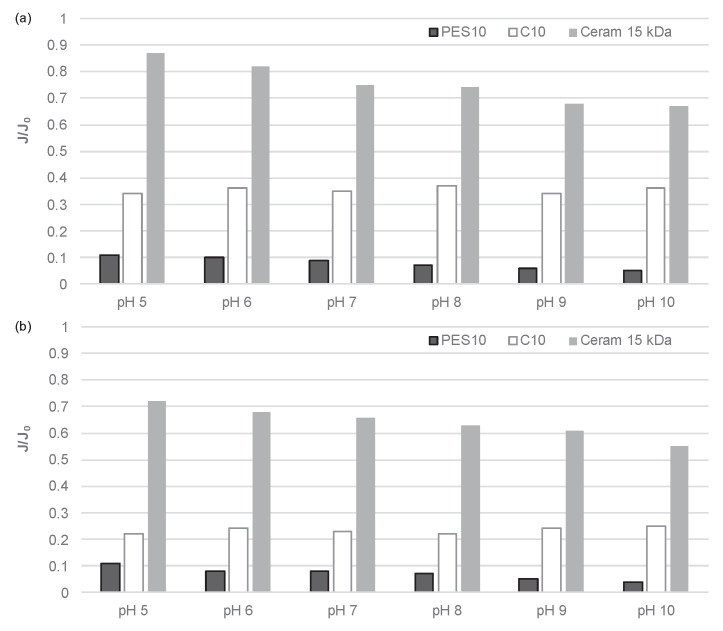
pH dependence of the relative permeability of polymeric and ceramic membranes for integrated process: sedimentation/filtration/coagulation/sedimentation/UF of the liquid fraction of (**a**) municipal and (**b**) agricultural digestates.

Furthermore, a comparison was conducted between the fouling intensities of three UF membranes: one ceramic membrane with a cut-off of 15 kDa and two polymeric membranes with a comparable cut-off value of 10 kDa, under conditions of altered pH of the filtered solution (Figure 7). It was observed that an increase in pH value resulted in an increase in the intensity of membrane fouling for both test solutions, including those comprising organic membranes made of polyethersulfone and inorganic membranes. On the contrary, no change in the intensity of membrane fouling was observed for regenerated cellulose membranes. It can, therefore, be concluded that the use of membranes made of highly hydrophilic materials, such as regenerated cellulose, can, to some extent, reduce the influence of the physicochemical properties of the filtered solution on membrane fouling.

### 3.2. Separation Properties of Membranes

**Figure 8:**
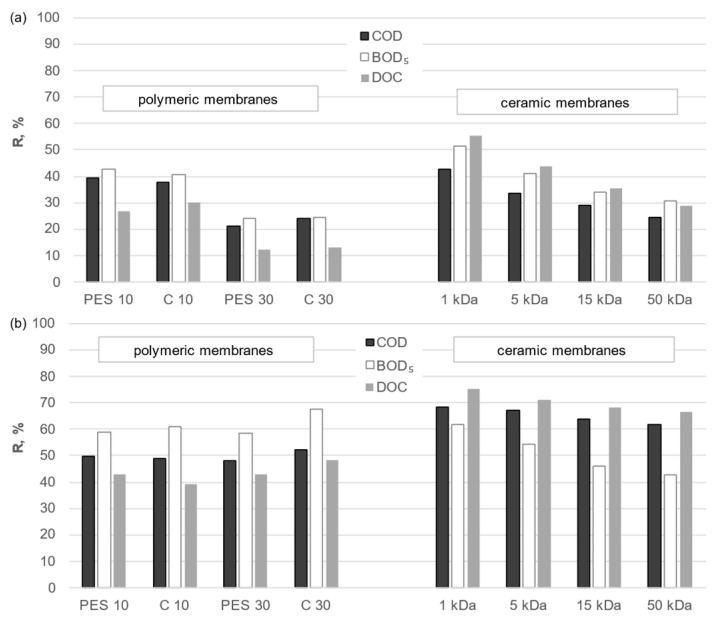
Effectiveness of DOC removal and reduction in BOD_5_ and COD when conducting (**a**) UF and (**b**) integrated process: sedimentation/filtration/coagulation/sedimentation/UF of the liquid fraction of municipal digestate using polymeric and ceramic membranes.

**Figure 9:**
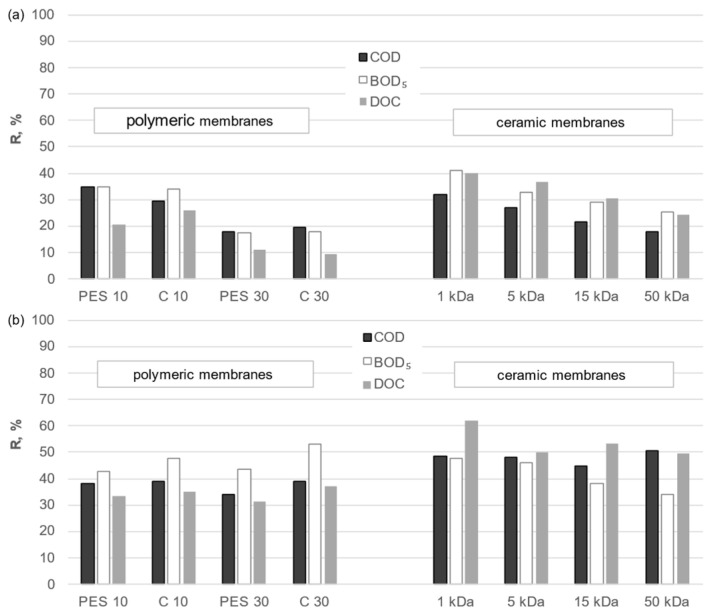
Effectiveness of DOC removal and reduction in BOD_5_ and COD when conducting (**a**) UF and (**b**) integrated process: sedimentation/filtration/coagulation/sedimentation/UF of the liquid fraction of agricultural digestate using polymeric and ceramic membranes.

In order to evaluate the efficacy of the membranes under investigation, the impact of membrane type on the separation efficiency of organic compounds from both solutions was subjected to analysis. A comparison of the purification efficiencies of the UF process and the integrated process combining conventional purification processes with the UF process on organic and inorganic membranes is presented in Figure 8 and Figure 9. The cut-off value stated by the manufacturer is often a determining factor in the selection of the appropriate membrane. Therefore, it was deemed appropriate to refer to this value when comparing the two membrane types. The results of the analysis indicate that the removal efficiency of organic macromolecules was markedly higher for ceramic membranes than for polymeric membranes with comparable cut-off values. To illustrate, the use of a ceramic membrane with a cut-off value of 15 kDa in a standalone UF process for the treatment of municipal digestate resulted in retention rates of 35%, 34%, and 29% for DOC, BOD_5_, and COD, respectively. This equates to a removal of 2910 to 1880 g C/m^3^ and a reduction from 1370 to 1110 g O_2_/m^3^. The PES 10 membrane demonstrated comparable performance at 25, 28, and 11% (reduction to 2190 g C/m^3^, 1370 g O_2_/m^3^, and 5220 g O_2_/m^3^, respectively). The results demonstrated that the PES 10 membrane exhibited the highest removal efficiency at 26, 28, and 11% (2160 g C/m^3^ and 5250 g O_2_/m^3^, respectively), while the C 10 membrane demonstrated the lowest removal efficiency at the same concentrations (1385 g O_2_/m^3^ and 5220 g O_2_/m^3^, respectively). It can, thus, be concluded that the material of the polymeric membrane employed, irrespective of the process utilized, had no discernible impact on the separation properties of the membranes under examination. Both polyethersulfone and regenerated cellulose yielded permeates with comparable organic content.

The ceramic membrane with a cut-off of 1 kDa yielded the most favorable results in the standalone membrane separation process. The separation efficiency for liquid municipal digestate was DOC at 55%, BOD_5_ at 51%, and COD at 43% (reduction to 1300 g C/m^3^, 930, and 3365 g O_2_/m^3^, respectively). On the contrary, the results for liquid agricultural digestate were 40%, 41%, and 32% (reduction to 13,830 g C/m^3^, 7265, and 26,200 g O_2_/m^3^, respectively). The contaminant removal of all membranes analyzed was determined by the sieve mechanism. The ratio of contaminant size to membrane pore diameter had a significant effect on removal efficiency, as discussed in [30].

Similar outcomes were observed when membrane separation was coupled with conventional treatment processes. The retention factors for DOC, BOD_5_, and COD were higher when ceramic membranes were employed than when polymeric membranes were used, irrespective of the nature of the solution undergoing treatment.

In both the standalone UF process and the integrated process, membrane cut-off resolution emerged as a significant consideration. As the cut-off point and, consequently, the average pore size increased, the quality of the treated solution’s permeate declined. The utilization of membranes with a more compact structure enabled the attainment of permeate with a reduced organic content. Furthermore, the sedimentation process conducted at the pretreatment stage prior to coagulation facilitated the partial clarification of the solution by reducing the concentration of larger particles in the analyzed solution. In addition, the suspended fraction was eliminated by filtration. Therefore, the final quality of the investigated digestate was determined by the combination of the efficiency of the conventional purification processes applied and the separation properties of the membrane. The separation mechanism in the analyzed integrated process is a combination of the coagulation effect of FeCl_3_⋯6 H_2_O of the presuspended solution and the sieve mechanism on the membranes tested.

The specific type of liquid digestate fraction subjected to analysis is also of considerable importance. It has been demonstrated that the efficiency of the separation of organic pollutants from liquid digestate is significantly influenced by the composition of the treated solution, regardless of whether the membrane is organic or inorganic. Despite the comparable content of organic compounds in the liquid fraction of agricultural-derived digestate and the liquid fraction of municipal-derived digestate, the former did not contain the same amount of other organic and inorganic pollutants. It was found that the retention rates of DOC, BOD_5_, and COD during the treatment of municipal digestate were significantly higher than those observed for agricultural digestate. This trend was observed for all membranes tested. The presence of inorganic ions in liquid agricultural digestate has been shown to influence both the spatial configuration of organic particles and the surface charge, which can facilitate the transport of organic substances through the membrane. This highlights the need for further research and verification of the results obtained for different types of liquid digestate fractions.

**Figure 10:**
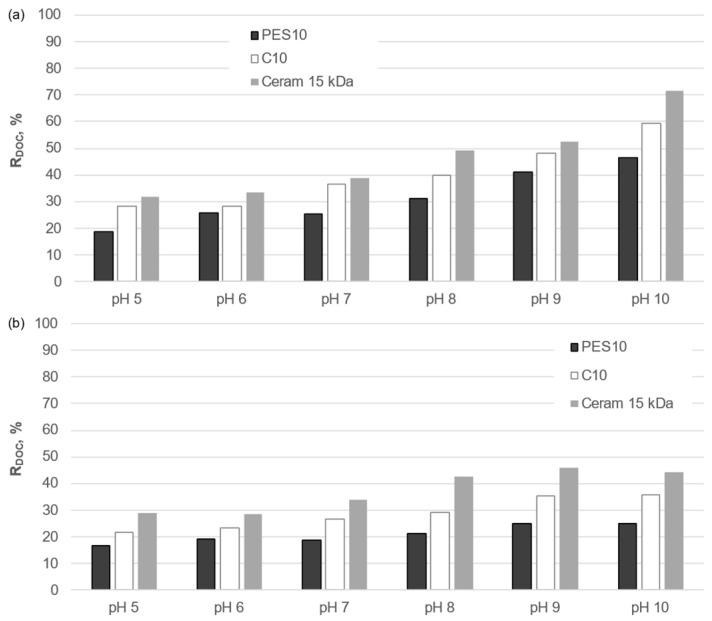
Influence of solution pH on the degree of DOC reduction on organic and inorganic membranes during treatment of the liquid fraction of municipal digestate by (**a**) UF and (**b**) integrated: sedimentation/filtration/coagulation/sedimentation/UF processes.

**Figure 11:**
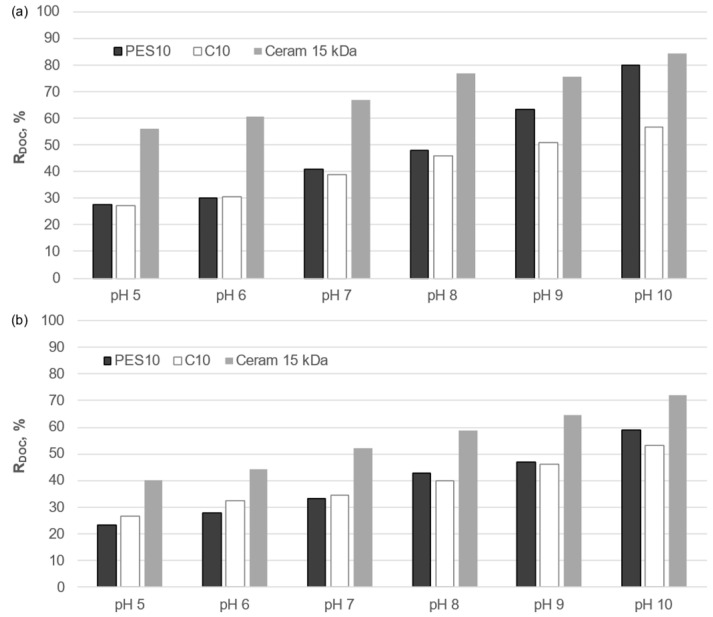
Influence of solution pH on the degree of DOC reduction on organic and inorganic membranes during treatment of the liquid fraction of agricultural digestate by (**a**) UF and (**b**) integrated: sedimentation/filtration/coagulation/sedimentation/UF processes.

As was the case in the determination of the transport properties of the membranes, a comparison was made between the effect of the solution reaction on the separation properties of polymeric and ceramic membranes with comparable cut-off values (Figure 10 and Figure 11). The results of the analysis demonstrate a notable influence of the pH of the test solution on the purification of the liquid fraction of municipal and agricultural digestate on both types of membrane, regardless of the purification process used. An increase in the pH value within the range of 5 to 10 was observed to result in increased effectiveness of the reduction in DOC for both organic and inorganic membranes. The lowest retention was observed at pH 5, while an increase in the pH value in the analyzed range resulted in an improvement in the separation efficiency of organic macromolecules for both organic and inorganic membranes. This effect is attributed to the fact that at pH = 5, organic macromolecules are poorly dissociated, resulting in a relatively small spatial dimension and enhanced penetration through the membranes. As the pH value of the solution increases, the phenolic and carboxyl functional groups of organic compounds dissociate, resulting in their larger spatial dimension and easier retention on the membrane. Furthermore, the adsorption of anions from the solution onto the pore walls of the membrane is also possible [31], which results in a reduction in the membrane pore diameter and stronger electrostatic interactions between organic macroanions and the membrane.

## 4. Conclusions

The suitability of the UF process, either alone or in combination with conventional processes to form an integrated process, has been confirmed by studies for the treatment of different types of liquid digestate (from an agricultural biogas plant and from a biogas plant treating the organic fraction of municipal waste). Given the fundamental differences between the two types of digestate, it was imperative to conduct dedicated research on each. The findings demonstrate that the treatment of liquid digestate in the aforementioned manner facilitates the recovery of water, which, following purification, can be utilized in agricultural applications, including the preparation of fertilizers or irrigation. A comparative analysis of the transport and separation properties of membranes comprising organic and inorganic materials employed for the purification of liquid digestate yielded the following conclusions:

1. Polymeric membranes are characterized by higher hydraulic permeability values compared to ceramic membranes.
2. Permeate fluxes during membrane filtration (standalone or preceded by pretreatment) of the liquid fraction of agricultural digestate were lower than those measured for the liquid fraction of municipal digestate.
3. Inorganic membranes are more susceptible to fouling when used in a standalone ultrafiltration (UF) process, whereas organic membranes are more susceptible in an integrated process.
4. The removal efficiency of organic macromolecules was significantly higher when using ceramic membranes than polymeric membranes with a comparable cut-off value.
5. For both types of membranes tested, an increase in the pH value of the solution resulted in an increase in the intensity of membrane fouling, as well as an improvement in the separation efficiency of organic macromolecules.

## Figures and Tables

**Table 1:** Composition of liquid digestate fraction from municipal and agricultural biogas plants.

	Liquid Digestate Fraction from the Municipal Waste Biogas Plant	Liquid Digestate Fraction from the Agricultural Biogas Plant
pH	7.17	7.21
Conductivity, mS/cm	25.21	14.95
Total suspended solids, mg/dm^3^	670	3950
Chemical oxygen demand (COD), mg O_2_/dm^3^	29,360	38,595
5-day biochemical oxygen demand (BOD_5_), mg O_2_/dm^3^	8690	12,320
Dissolved organic carbon (DOC), mg C/dm^3^	8650	23,070
Na, mg/dm^3^	487.2	521.3
K, mg/dm^3^	1678.4	1966.5
Ca, mg/dm^3^	89.2	104.7
Mg, mg/dm^3^	672.2	101.9
Fe, mg/dm^3^	6.2	15.9
Mn, mg/dm^3^	4.4	1.5
Cu, mg/dm^3^	0.230	0.545
Zn, mg/dm^3^	1.434	3.977
Hg, mg/dm^3^	0.0040	0.0029
Co, mg/dm^3^	0.156	0.069
Ni, mg/dm^3^	0.320	0.147

**Table 2:** The analytical procedures conducted and the instrumentation employed.

Examined Parameter	Method	Apparatus
pH	Potentiometric method	Digital multimeter HQ40D with IntelliCAL^TM^ PHC 101 electrode (Hach, Ames, IA, USA)
Conductivity	Conductometric method	
Total suspended solids	Weight-based method	-
COD	Bichromate method	
BOD_5_	Dilution method	
DOC	NPOC high temperature oxidation method; thermal method	Hach IL550 carbon analyzer (Hach, Ames, IA, USA)
Na, K	Ion chromatography method	Thermo Scientific Dionex Aquion ion chromatograph with a conductometric detector for anions or cations analysis (Thermo Fisher Scientific, Waltham, MA, USA)
Ca, Mg	Titration method	-
Fe, Mn	Spectrophotometric method	Shimadzu UV-VIS 1800 (Shimadzu Corporation, Kyoto, Japan)
Cu, Zn, Co, Ni	Atomic absorption spectroscopy (ASA) with flame atomization	Atomic absorption spectrometer iCE 3500 (Thermo Fisher Scientific, Waltham, MA, USA)
Hg	Atomic absorption spectroscopy (ASA)—selective for Hg with concentration by amalgamation	AMA 254 mercury analyzer (Leco Corporation, St. Joseph, MI, USA)

**Table 3:** Characteristics of the membranes used in the experiments [28,29].

Membrane Type		Membrane Material	Cut-Off	Max. Press., MPa	Max Temp., °C	pH Range	Active Filtration Area, cm^2^
Flat polymeric membranes							
PES 10 kDa	UF	Polyethersulfone	10 kDa	-	95	1–14	38.5
PES 30 kDa	UF		30 kDa				
C 10 kDa	UF	Regenerated cellulose	10 kDa		55	1–11	
C 30 kDa	UF		30 kDa				
Flat ceramic membranes							
Ceram 1 kDa	FINE UF	TiO_2_	1 kDa	0.4	350	2–14	56
Ceram 5 kDa	FINE UF		5 kDa				
Ceram 15 kDa	UF	ZrO_2_	15 kDa			0–14	
Ceram 50 kDa	UF		50 kDa				

## Data Availability

The data that support the findings of this study are available from the corresponding author upon reasonable request.
